# Editorial: Epilepsy and Alzheimer's disease: shared pathology, clinical presentations, and targets for treatment

**DOI:** 10.3389/fneur.2024.1441996

**Published:** 2024-08-13

**Authors:** Beth Leeman-Markowski, Jeannie Chin, Dominique Leitner, Keith Vossel

**Affiliations:** ^1^Neurology Service, VA New York Harbor Healthcare System, New York, NY, United States; ^2^Department of Neurology, Comprehensive Epilepsy Center, New York University Langone Health, New York, NY, United States; ^3^Department of Neuroscience, Baylor College of Medicine, Houston, TX, United States; ^4^Department of Neurology, Center for Cognitive Neurology, New York University Langone Health, New York, NY, United States; ^5^Department of Neurology, Mary S. Easton Center for Alzheimer's Research and Care, David Geffen School of Medicine, University of California, Los Angeles, Los Angeles, CA, United States

**Keywords:** epilepsy, Alzheimer's disease, memory, seizures, cognition, dementia, tau, amyloid

While epilepsy incidence peaks in older adults (1, 2), the association between epilepsy and Alzheimer's disease (AD) extends beyond the increased risk of AD with age. Epilepsy and AD share clinical manifestations, with approximately 50% of epilepsy patients demonstrating cognitive dysfunction (3, 4) and prevalence estimates of seizures in AD ranging widely from 1.5 to 75% (5, 6). Epilepsy and AD can also have similar pathological findings, with beta-amyloid and tau accumulation, and selective vulnerability of the hippocampus, in both disorders (7, 8). Many questions remain unanswered, however, regarding similarities and differences in cognitive profiles, identification of biomarkers, underlying mechanisms, and treatment implications. Articles in this collection address these fundamental questions.

## Clinical presentations

Risks of developing epilepsy and dementia are bidirectional, with an estimated two-fold risk of one disorder in the setting of the other (9). Hence, we must know when to suspect a dual diagnosis. Reyes et al. described cognitive phenotypes of late onset epilepsy (LOE), finding that 62.5% declined in cognitive performance over a median of 4 years. The authors concluded that developing seizures in older age can accelerate cognitive decline. Performance decrements, however, may be challenging to distinguish from AD. Liu and Barr highlighted differing patterns of memory deficits corresponding to cell loss in different hippocampal subfields in LOE and AD. With early neuronal loss in the dentate gyrus and CA1/CA3 regions in temporal lobe epilepsy (TLE), there is corresponding difficulty with separation of details, and association and consolidation between present and past events, with relatively spared encoding and retrieval. In contrast, AD involves early cell loss in the entorhinal cortex, impairing all stages of memory formation and retrieval. The authors proposed that in early stages, TLE and AD could be distinguished based on these differing patterns of memory dysfunction.

## Biomarkers

Liu and Barr and Lu et al. reviewed similarities between AD and epilepsy, including amyloid and tau pathology. Adults with epilepsy can exhibit early AD pathology, including lower Aβ42 in cerebrospinal fluid (CSF) and hyperphosphorylated tau in the temporal lobes (10, 11). AD patients with comorbid epilepsy have greater abnormalities in CSF Aβ42, total tau, and phosphorylated tau than AD patients without epilepsy (12). Hickman et al. recommended that all patients with late onset epilepsy of unknown cause (LOEU) have an evaluation for preclinical or prodromal AD and categorized LOEU based on presence or absence of amyloid and tau biomarkers. These categories will likely become more refined as we develop more comprehensive biomarkers of seizure-associated proteinopathies, including alpha-synuclein, TDP-43, and immune factors.

Martin and Leeman-Markowski proposed a mechanism by which hyper-phosphorylated tau and neurofibrillary tangles accumulate in epilepsy, resulting from an imbalanced endoplasmic reticulum stress response, inflammatory signaling, and a failed “last ditch effort” of amyloid-beta to revert the cell to programmed cell death. They presented a hypothesis of tau phosphorylation as an acute neuroprotective response to seizures that may transition to an injurious process when these pathways are chronically activated by repeated seizures.

Leitner et al. examined proteins within the choroid plexus (13, 14) of human post-mortem tissue. They identified protein differences in the choroid plexus of AD compared to controls, associated with a shift from glucose-mediated energy production to fatty acid beta-oxidation activation and glycolysis inhibition, coupled with activated branched-chain amino acid degradation. Greater variability and fewer protein differences were evident in the epilepsy group compared to controls, but similar trends in protein changes were present in epilepsy and AD. Proteomics of the choroid plexus and other brain regions (15) may inform future mechanistic and therapeutic studies.

## Genetics

Epigenetic regulation of gene expression can translate intermittent seizures to long-lasting cognitive changes. The neuronal activity-induced transcription factor ΔFosB is robustly increased in the dentate gyrus in AD and correlates with cognitive impairment (16). Although seizure-induced ΔFosB accumulation occurs in TLE (16, 17), whether it is associated with cognitive deficits in epilepsy is unknown. Fu et al. found increased ΔFosB expression in pediatric epilepsies that was inversely related to IQ in patients with intellectual disabilities. Thus, ΔFosB expression may contribute to cognition in a range of epilepsy syndromes.

Multiple ΔFosB target genes in the hippocampus play critical roles in calcium handling and synaptic plasticity, which may explain why their suppression by ΔFosB leads to cognitive deficits (16–18). However, prolonged ΔFosB expression may also enable neuroprotective and homeostatic pathways. In Clasadonte et al., prolonged ΔFosB reduction exacerbated neuroinflammatory pathways in mouse models of epilepsy. Their newly developed shRNA tool for reducing ΔFosB expression was effective and long-lasting, revealing that ΔFosB maintains neuroprotection, in part by limiting astrocyte and microglial engagement in neuroinflammation. These results are consistent with prior studies demonstrating that prolonged blockade of ΔFosB exacerbates seizures and memory deficits in an AD mouse model (19). Together, these data reveal how engagement of ΔFosB by recurrent seizures contributes to long-lasting impacts on hippocampal gene expression and function.

## Treatment

Lu et al. provided an overview of AD medication effects on seizure threshold, which can guide clinicians when selecting individualized treatments. We must also better understand anti-seizure medications (ASMs) in the context of AD with epileptiform activity. Lehmann and Barker-Haliski evaluated acute ASM potency and tolerability in a presenilin-2 (PSEN2) knockout (KO) early onset-AD mouse model in comparison to wild type controls, using a 6-Hz limbic seizure test. Acute potency and tolerability across multiple ASMs were altered with PSEN2 loss, providing support for targeted ASM therapy analyses in familial early-onset AD patients.

Overlapping clinical presentations and neuropathological changes of AD and epilepsy could lead to shared treatments (20–24). Further, interictal epileptiform discharges (IEDs), may serve as a target for treatment in AD. Lu et al. highlighted that seizures and IEDs in AD are associated with accelerated cognitive decline and that ASMs may improve cognitive function in AD patients with epileptiform activity, which is most commonly seen in sleep (25–28). Lemus and Sarkis advised a measured approach to considering ASMs in AD patients with IEDs, taking into account the patient's age and the frequency, morphology, and other characteristics of the epileptiform activity.

## Related dementias

The bidirectional risk of epilepsy and dementia is not limited to AD (29). Vicente et al. noted the increased risk of epilepsy in dementia with Lewy bodies (DLB). Many of the same pathological changes and pathways are implicated in AD and DLB, including glutamate transporter imbalance, cholinergic neuron degeneration, mechanistic target of rapamycin (mTOR) overactivation, and disruption of glial immunoinflammatory function, such that mechanistic insights into epileptic activity in one disease could be informative for the other.

## Conclusion

The studies highlighted in this collection contribute to a greater understanding of the relationships between epilepsy and AD, with the hope of improving diagnosis and identifying effective treatments, so patients can have improved cognition.

## Author contributions

BL-M: Conceptualization, Funding acquisition, Project administration, Writing – original draft, Writing – review & editing. JC: Writing – original draft, Writing – review & editing. DL: Writing – original draft, Writing – review & editing, Funding acquisition. KV: Funding acquisition, Writing – original draft, Writing – review & editing.
